# Global prevalence of prediabetes in children and adolescents: A systematic review and meta‐analysis

**DOI:** 10.1111/1753-0407.13291

**Published:** 2022-07-05

**Authors:** Chengyi Han, Qing Song, Yongcheng Ren, Xinyu Chen, Xuesong Jiang, Dongsheng Hu

**Affiliations:** ^1^ The First Affiliated Hospital of Henan University of CM Zhengzhou Henan China; ^2^ School of Public Health, Shenzhen University Health Science Center Shenzhen Guangdong China; ^3^ The Medical Collage of Huanghuai University Zhumadian Henan China; ^4^ School of Public Health, Southwest Medical University Chengdu Sichuan China

**Keywords:** adolescent, meta‐analysis, prediabetes, prevalence, systematic review, 青少年, 荟萃分析, 前期糖尿病, 患病率, 系统回顾

## Abstract

**Background:**

Prediabetes is a pivotal risk factor for developing diabetes. This meta‐analysis was performed to assess the global prevalence of childhood prediabetes.

**Methods:**

A systematic search was conducted for studies of prediabetes prevalence in the general pediatric population from inception until December 2021. Random‐effects meta‐analysis was used to combine the data. Variations in the prevalence estimates in different subgroups (age group, sex, setting, investigation period, body mass index [BMI] group, family history of diabetes, diagnosis criteria, World Health Organization [WHO] and World Bank [WB] regions) were examined by subgroup meta‐analysis.

**Results:**

A total of 48 studies were included in the meta‐analysis. The pooled prevalence was 8.84% (95% CI, 6.74%‐10.95%) for prediabetes in childhood. Subgroup meta‐analyses showed that the prevalence was higher in males than females (8.98% vs 8.74%, *P* < .01), in older compared to younger children (7.56% vs. 2.51%, *p* < 0.01), in urban compared to rural areas (6.78% vs. 2.47, *p* < 0.01), and higher in children with a family history of diabetes than in those without such a history (7.59% vs. 6.80%, *p* < 0.01). We observed an upward trend in prediabetes prevalence from 0.93% to 10.66% over past decades (*p* < 0.01). The pooled prevalence increased from 7.64% to 14.27% with increased BMI (*p* < 0.01). Pooled prevalence was the lowest for criterion A among different diagnosis criteria (*p* < 0.01). For WHO and WB regions, the European Region and high‐income countries yielded the lowest pooled prevalence (*p* < 0.01).

**Conclusions:**

Elevated prediabetes prevalence in childhood reaches an alarming level. Intensive lifestyle modification is needed to improve the prediabetes epidemic.

## Graphical abstract

**Highlights**

- There was a growing trend in childhood prediabetes prevalence worldwide with time and BMI.
- Higher prevalence was observed for males than females, older than younger children, urban than rural areas, and children with family histories of diabetes than those without such a history.

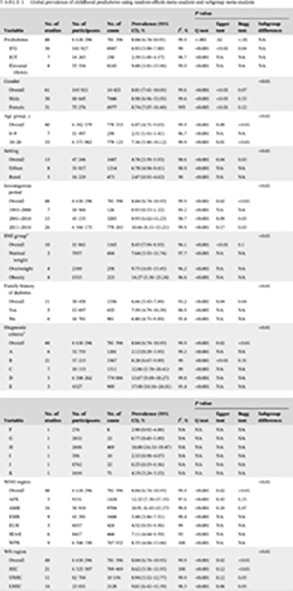

## 摘要

**背景:**

前期糖尿病是糖尿病发生的关键危险因素。这项荟萃分析旨在评估全球儿童前期糖尿病的患病率。

**方法:**

对从研究开始到2021年12月的普通儿科人群中前期糖尿病患病率的研究进行了系统搜索。采用随机效应荟萃分析数据。通过亚组meta分析检查不同亚组(年龄组、性别、环境、调查周期、体重指数(BMI)、糖尿病家族史、诊断标准、世界卫生组织和世界银行地区)患病率估计值的差异。

**结果:**

共有48项研究被纳入meta分析。儿童前期糖尿病的综合患病率为8.84% (95% CI, 6.74%‐10.95%)。亚组meta分析显示，男性患病率高于女性(8.98% vs. 8.74%，P<0.01)，大龄儿童患病率高于低龄儿童(7.56% vs. 2.51%，P<0.01)，城市儿童患病率高于农村儿童(6.78% vs. 2.47,P<0.01)，有糖尿病家族史儿童患病率高于无糖尿病家族史儿童(7.59% vs. 6.80%，P<0.01)。我们观察到在过去的几十年中，糖尿病前期患病率从0.93%上升到10.66% (P<0.01)。随着BMI的增加，合并患病率从7.64%上升到14.27%(P<0.01)。在不同诊断标准中，A标准(FPG = 6.1‐7.0 mmol/L)合并患病率最低(P<0.01)。就世卫组织和世界银行地区而言，欧洲区域和高收入国家的总患病率最低(P<0.01)。

**结论:**

儿童前期糖尿病患病率升高达到了一个惊人的水平。需要加强生活方式的改变来改善前期糖尿病的流行。

## 1 INTRODUCTION

Diabetes is an important predictor of mortality, morbidity, and health system costs in the world. It is estimated that the number of adults across the globe with diabetes will increase from 432 million (9.3% of the population) in 2019 to 700 million adults (10.9% of the population) by 2045.^1^ It is not only highly prevalent in adults; the incidence of diabetes has also been increasing among young people.^2^ In addition, the progress of diabetes in young participants could be faster and more disruptive than in participants with later onset of the disease, causing early morbidity and reduced quality of life.^3^, ^4^ The early presentation of prediabetes and diabetes in children raises the possibility of an accelerated pathophysiologic process in the young.^5^

Given that prediabetes has a pivotal role in the risk of developing diabetes^6^ and that diabetes incidence has increased markedly over recent decades, reliable estimates of the prevalence of prediabetes should serve as the rationale for comprehensive interventions to increase the rate of reversion from prediabetic states to normal glucose tolerance.^7^, ^8^ Despite a review assessing the prevalence of prediabetes in childhood,^9^ its prevalence has not been estimated at the global level. This systematic review and meta‐analysis therefore synthesizes data on the global prevalence of prediabetes (impaired fasting glucose [IFG], impaired glucose tolerance [IGT], increased glycosylated hemoglobin [HbA1c], or combinations of these) among children and adolescents. The factors potentially associated with childhood prediabetes were also explored to effectively delay and even prevent the development of prediabetes.

## 2 MATERIALS AND METHODS

### 2.1 Data source and searches

Focusing on studies published in English up until December 2021, two researchers (C.H. and Q.S.) independently conducted a systematic search of PubMed, Embase, and Web of Science by using a combination of search terms related to prediabetes (Prediabetic State OR Hyperglycemia OR impaired fasting glucose OR impaired glucose tolerance OR pre‐diabetes or prediabetes OR borderline diabetes), prevalence (prevalence OR epidemiology), and children (children OR adolescents). We did not obtain gray literature or unpublished research to inform this manuscript. To complement our database, the reference lists of included articles were also searched. Search strategies are outlined in Table S1 in Appendix S1. This study was conducted in accordance with the preferred reporting guidelines for systematic reviews and meta‐analyses (PRISMA).

### 2.2 Study selection

We included (1) studies that reported a community‐based children or adolescents (≤20 years of age) population; (2) studies that reported the prevalence of prediabetes (IFG, IGT, or elevated HbA1c) or data to calculate them; (3) cross‐sectional studies or cohort studies; and (4) those for which definitions of prediabetes were explicitly provided. We included studies that were conducted with both adults and children where the prevalence data of prediabetes could be disaggregated for the pediatric group. Where multiple articles reported the same investigation, the one giving more complete details or with the largest sample size was included, but when different aspects or subgroups of the same survey were separately reported in different articles, they were selected. We excluded (1) reviews, case reports, editorials, guidelines, or letters to the editor; (2) case‐control studies; (3) and surveys that included participants with any disease or complication. If the epidemiological survey lasted more than a year, the year of study completion was used as the time variable. We emailed corresponding authors of the included studies to acquire the years of investigation for the included studies. If the survey time was not obtained after the correspondence, we imputed it by subtracting 4 years from the year of publication based on the mean time difference of the years of investigation and publication for which data are provided in Table S2 in Appendix S1. After removing duplicates articles, the two researchers (C.H. and Q.S.) independently evaluated the retrieved studies to identify those that were relevant. Disagreements were resolved by consensus or by discussion with a third researcher (Y.R.).

### 2.3 Data extraction

Information on first author, publishing year, survey time, country, setting (urban versus rural), date source, response rate, age range, sample size, case, crude prevalence rate, and diagnosis criteria was independently extracted by two reviewers (C.H. and Q.S.) for each included study. For cohort studies, we extracted the data for the baseline assessment. The regions of study location were grouped by continent (African Region [AFR], Region of the Americas [AMR], South‐East Asia Region [SEAR], European Region [EUR], Eastern Mediterranean Region [EMR], and Western Pacific Region [WPR]) according to World Health Organization (WHO) criteria and income levels (low‐income countries: gross national income [GNI] per capita ≤US$1035; lower‐middle‐income countries [LMIC]: GNI per capita US$1036‐4045; upper‐middle‐income countries [UMIC]: GNI per capita US$4046‐12 535; high‐income countries [HIC]: GNI per capita ≥US$12536) using the World Bank [WB] data updated in 2019.^10^

### 2.4 Quality assessment

Two reviewers (C.H. and Q.S.) evaluated the quality of the included studies using the 10‐item tool developed by Hoy et al^11^ (Table S3 in Appendix S1). The total score was the sum of scores for each item, with 9 to 10, 6 to 8, and 0 to 5 representing high, moderate, and low quality, respectively. If reviewers' scores differed, they discussed them until consensus was reached.

### 2.5 Statistical analysis

Data are presented as percentages with 95% CI. Heterogeneity of studies was estimated by the Q test and *I* ^2^ index. We used a random‐effects model to calculate a pooled estimation of prediabetes prevalence. Subgroup meta‐analyses for childhood prediabetes were also conducted to explore the source of heterogeneity of included studies. As a rule, at least three studies had to be available per subgroup. Chi‐square and Cochran‐Mantel‐Haenszel tests were used to estimate differences among subgroups and the trend of pooled prevalence, respectively. When more than 10 studies were included in a single analysis, publication bias was assessed by the Egger linear regression and the Begg rank correlation tests. To detect single studies with disproportionate influence, we conducted a leave‐1‐out sensitivity analysis for each meta‐analysis. Statistical significance was set at *P* < .05. Analyses involved use of Stata 11.0 (StataCorp).

## 3 RESULTS

### 3.1 Literature search and study characteristics

**FIGURE 1:**
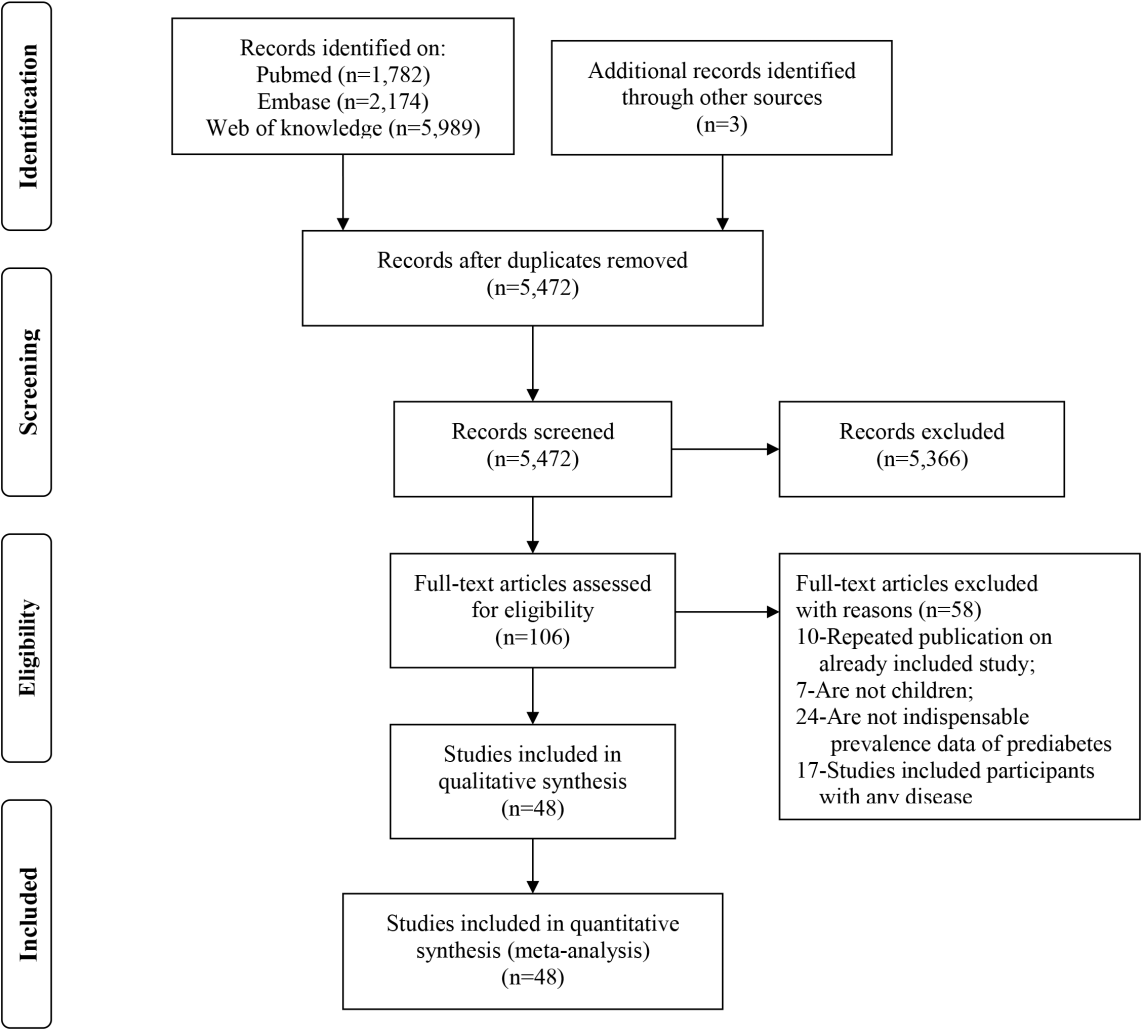
Flowchart of study selection.

Of the 9948 potentially relevant studies identified through the database search, 48 articles were included in the final analysis (Figure 1). The details of eligible studies are shown in Table S4 in Appendix S1. A total of 6 630 296 participants were included in the analysis. The total prevalence of prediabetes ranged from 0.25% to 23.47% over the previous decades. A total of 30 of the included studies presented the prevalence data of both sexes; 21 of 48 studies were conducted in the HIC, 11 in the UMIC, and 16 in the LMIC. In all, 16 studies were from the AMR, 9 from the EMR, 9 from the WPR, 6 from the SEAR, 5 from the AFR, and 3 from the EUR. All the included literature had a quality score of at least six.

### 3.2 Pooled and stratified prevalence of childhood prediabetes

Table 1 shows the overall results. Significant heterogeneity was found among studies (*I* ^2^ = 99.9, *p* < 0.01; Figure S1 in Appendix S1). A random‐effects meta‐analysis revealed a pooled prevalence of childhood prediabetes of 8.84% (95% CI, 6.74%–10.95%). The sensitivity analysis showed that the pooled prevalence varied from 8.53% (95% CI, 6.41%–10.65%) to 9.01% (95% CI, 7.04%–10.98%) after removing a single study at a time (Figure S2 in Appendix S1), but no single study had a disproportionate influence on the pooled prevalence. We found publication bias using Egger and Begg tests (Table 1). The pooled prevalence of different prediabetes phenotypes was also estimated using random‐effects models: 6.93% (95% CI, 5.98%–7.88%) for IFG, 2.59% (95% CI, 1.00%–4.17%) for IGT, and 9.88% (95% CI, 3.81%–15.96%) for elevated HbA1c.

**TABLE 1:** Global prevalence of childhood prediabetes using random‐effects meta‐analysis and subgroup meta‐analysis

Variable	No. of studies	No. of participants	No. of cases	Prevalence (95% CI), %	*I* ^2^, %	*P* value			
						Q test	Egger test	Begg test	Subgroup difference
Prediabetes	48	6 630 296	781 596	8.84 (6.74‐10.95)	99.9	<.001	.02	<.01	NA
IFG	36	141 927	6947	6.93 (5.98–7.88)	99	<0.001	<0.01	0.04	NA
IGT	7	14 265	230	2.59 (1.00–4.17)	96.7	<0.001	NA	NA	NA
Elevated HbA1c	8	55 554	8145	9.88 (3.81–15.96)	99.9	<0.001	NA	NA	NA
Gender									<0.01
Overall	61	143 921	14 423	8.81 (7.61–10.01)	99.6	<0.001	<0.01	0.07	
Male	30	68 645	7446	8.98 (6.96–11.01)	99.6	<0.001	<0.01	0.33	
Female	31	75 276	6977	8.74 (7.07–10.40)	995	<0.001	<0.01	0.12	
Age group, y									<0.01
Overall	40	6 582 579	778 353	6.87 (4.71–9.03)	99.9	<0.001	0.08	<0.01	
0–9	7	11 497	230	2.51 (1.61–3.41)	86.7	<0.001	NA	NA	
10–20	33	6 571 082	778 123	7.56 (5.40–10.12)	99.9	<0.001	0.01	<0.01	
Setting									<0.01
Overall	13	47 246	1687	4.76 (3.59–5.93)	98.6	<0.001	0.04	0.03	
Urban	8	31 017	1214	6.78 (4.94–8.61)	98.9	<0.001	NA	NA	
Rural	5	16 229	473	2.47 (0.93–4.02)	98	<0.001	NA	NA	
Investigation period									<0.01
Overall	48	6 630 296	781 596	8.84 (6.74–10.95)	99.9	<0.001	0.02	<0.01	
1993–2000	7	18 968	126	0.93 (0.53–1.32)	89.2	<0.001	NA	NA	
2001–2010	15	45 155	3205	8.93 (6.62–11.23)	98.7	<0.001	0.09	0.05	
2011–2018	26	6 566 173	778 265	10.66 (8.11–13.21)	99.9	<0.001	0.17	0.03	
BMI group^a^									<0.01
Overall	10	11 861	1165	8.43 (7.94–8.93)	96.1	<0.001	<0.01	0.1	
Normal weight	2	7957	684	7.64 (3.53–11.74)	97.7	<0.001	NA	NA	
Overweight	4	2389	258	9.75 (4.05–15.45)	96.2	<0.001	NA	NA	
Obesity	4	1515	223	14.27 (5.30–23.24)	96.8	<0.001	NA	NA	
Family history of diabetes									<0.01
Overall	11	30 458	1556	6.66 (5.43–7.89)	93.2	<0.001	0.04	0.04	
Yes	5	13 697	655	7.59 (4.79–10.39)	88.9	<0.001	NA	NA	
No	6	16 761	901	6.80 (4.71–8.89)	95.4	<0.001	NA	NA	
Diagnostic criteria^b^									<0.01
Overall	48	6 630 296	781 596	8.84 (6.74–10.95)	99.9	<0.001	0.02	<0.01	
A	6	32 753	1201	2.12 (0.29–3.95)	99.2	<0.001	NA	NA	
B	21	57 215	3367	8.28 (6.67–9.89)	99	<0.001	<0.01	0.31	
C	7	10 153	1511	12.06 (5.70–18.41)	99	<0.001	NA	NA	
D	5	6 506 262	774 006	13.67 (9.08–18.27)	99.8	<0.001	NA	NA	
E	3	4327	909	17.08 (10.16–24.01)	95.4	<0.001	NA	NA	
F	1	276	8	2.90 (0.92–4.88)	NA	NA	NA	NA	
G	1	2852	22	0.77 (0.45–1.09)	NA	NA	NA	NA	
H	1	2606	469	18.00 (16.52–19.47)	NA	NA	NA	NA	
I	1	396	10	2.53 (0.98–4.07)	NA	NA	NA	NA	
J	1	8762	22	0.25 (0.15–0.36)	NA	NA	NA	NA	
K	1	1694	71	4.19 (3.24–5.15)	NA	NA	NA	NA	
WHO region									<0.01
Overall	48	6 630 296	781 596	8.84 (6.74–10.95)	99.9	<0.001	0.02	<0.01	
AFR	5	9351	1424	12.32 (7.30–17.35)	97.6	<0.001	0.45	0.33	
AMR	16	56 910	9704	10.91 (6.45–15.37)	99.8	<0.001	0.18	0.47	
EMR	9	43 391	1668	5.48 (3.46–7.51)	99.4	<0.001	NA	NA	
EUR	3	6037	424	4.52 (0.53–9.56)	99	<0.001	NA	NA	
SEAR	6	8417	444	7.11 (4.64–9.59)	95	<0.001	NA	NA	
WPR	9	6 506 190	767 932	8.55 (4.04–13.06)	100	<0.001	NA	NA	
WB region									<0.01
Overall	48	6 630 296	781 596	8.84 (6.74–10.95)	99.9	<0.001	0.02	<0.01	
HIC	21	6 525 507	769 469	8.62 (5.30–11.95)	100	<0.001	0.12	<0.01	
UMIC	11	82 704	10 156	8.94 (5.12–12.77)	99.9	<0.001	0.12	0.05	
LMIC	16	23 031	2128	9.01 (6.42–11.59)	98.5	<0.001	0.06	0.05	

Abbreviations: AFR, African Region; AMR, Region of the Americas; BMI, body mass index; EMR, Eastern Mediterranean Region; EUR, European Region; FPG, fasting plasma glucose; HbA1c, glycosylated hemoglobin; HIC, high‐income countries; IFG, impaired fasting glucose; IGT, impaired glucose tolerance; LMIC, lower‐middle‐income countries; NA, not available; OGTT, oral glucose tolerance test; SEAR, South‐East Asia Region; UMIC, upper‐middle‐income countries; WB, World Bank; WHO, World Health Organization; WPR, Western Pacific Region.

^a^ International norms from the 2007 WHO with age (to the nearest 1 month) and gender‐specific BMI; BMI cutoffs were the following: normal, BMI ≥ −2 SD and ≤ 1 SD; overweight, BMI > 1 SD; obesity, BMI > 2 SD (SD was standard deviation of the BMI *z* scores).

^b^ The diagnostic criteria for prediabetes were as follows: A, FPG = 6.1‐7.0 mmol/L; B, FPG = 5.6–7.0 mmol/L; C, FPG = 5.6–7.0 mmol/L or 2‐h OGTT = 7.8–11.1 mmol/L; D, FPG = 5.6–7.0 mmol/L or HbA1c = 5.7%–6.4%; E, HbA1c = 5.7%–6.4%; F, FPG = 6.1–7.0 mmol/L or 2‐h OGTT = 7.8–11.1 mmol/L; G, FPG = 6.1–7.0 mmol/L or HbA1c > 6.0%; H, FPG = 5.6–7.0 mmol/L or 2‐h OGTT = 7.8–11.1 mmol/L or HbA1c = 5.7%–6.4%; I, HbA1c = 6.0%–6.4%; J, FPG <6.7 mmol/L or 2‐h OGTT = 6.7–10.0 mmol/L; K, FPG ≥ 5.6 mmol/L or random plasma glucose ≥7.8 mmol/L.

The results of subgroup analyses are also shown in Table 1. The prevalence among children was higher in males than females (8.98% [95% CI, 6.96%–11.01%] versus 8.74% [95% CI, 7.07%–10.40%]). Participants aged 10 to 20 years had a higher prevalence than those aged 0 to 9 years (7.56% [95% CI, 5.40%–10.12%] versus 2.51% [95% CI, 1.60%–3.41%]). The prevalence was greater in urban areas than in rural areas (6.78% [95% CI, 4.94%–8.61%] versus 2.47% [95% CI, 0.93%–4.02%]). The prevalence increased from 7.64% (95% CI, 3.53%–11.74%) to 14.27% (95% CI, 5.30%–23.24%) with body mass index (BMI) (*p* < 0.01). The prevalence for young participants has continued to increase, rising from 0.93% (95% CI, 0.53%–1.32%) to 10.66% (95% CI, 8.11%–13.21%) during the past decades. Compared with prediabetes prevalence among children with no family history of diabetes, prediabetes prevalence in children with a family history of diabetes was higher (7.59% [95% CI, 4.79%–10.38%] versus 6.80% [95%CI, 4.71%–8.89%]). Pooled prevalence was the highest for criterion E (17.08% [95% CI, 10.16%–24.01%]) and the lowest for criterion A (2.12% [95% CI, 0.29%–3.95%]). AFR yielded the highest pooled prevalence was (12.32% [95% CI, 7.30%–17.35%]) and EUR the lowest (4.52% [95% CI, 0.53%–9.56%]). A difference in childhood prevalence was also noted in different WB regions; LMIC (9.01% [95% CI, 6.42%–11.59%]) and UMIC (8.94% [95% CI, 5.12%–12.77%]) children had substantially higher prevalence estimates than children from HIC (8.62% [95% CI, 5.30%–11.95%]).

## 4 DISCUSSION

In this study, we describe the global prevalence of childhood prediabetes based on published data from1996 to 2021. Over that period, a rapidly growing trend in childhood prediabetes prevalence was observed. The prevalence increased with BMI and was higher in males than in females, in older compared to younger children, in urban areas compared to rural areas, and in children with family histories of diabetes compared to those without such a history.

Consonant with a national Korean study,^12^ we concluded an increase in the prevalence of childhood prediabetes at the global level. The increase in prediabetes prevalence worldwide may be associated with several factors. A positive association between prevalence of childhood prediabetes and age was observed in our study, which is consistent with a national investigation in Saudi Arabia.^13^ This finding indicates that older age may be a risk factor for childhood prediabetes. The development of prediabetes was seen more frequently in boys than in girls,^14^ a finding we also observed in the meta‐analysis. The differences in obesity^15^, ^16^ and hormone levels^17^ between males and females may explain the higher prediabetes prevalence we found in males. Our study demonstrated an upward trend in prediabetes prevalence in children with increased BMI. This finding is consistent with previous studies^18^, ^19^, ^20^ showing that obesity may be a risk factor for prediabetes, although one study found no relationship between obesity and prediabetes.^21^ The finding of a lower prevalence of prediabetes in rural areas compared with that in urban areas in this study is consistent with published data in China^22^ and Vietnam.^23^ Aging and growing obesity levels that accompany urbanization may therefore represent precursors to a global prediabetes epidemic. In another study, prevalence of IFG was 88% for those with family history compared to 1.9% for those without,^24^ similar to our study and the Cameron County Hispanic Cohort data.^25^ For WHO and WB regions, EUR and HIC yielded the lowest pooled prevalence. Differences in dietary habits, lifestyle, and sociodemographic factors among residents of different economic regions may explain these findings. The lowest pooled prevalence with criterion A may be explained by the highest cutoff point of fasting plasma glucose in this criterion compared with the other criteria.

### 4.1 Strengths and limitations

The current systematic review and meta‐analysis systematically synthesized the global prevalence of childhood prediabetes. Data were analyzed according to a common protocol, while the characteristics and quality of data sources were rigorously verified through repeated checks by two reviewers (Q.S. and C.H.). Moreover, the study provides further strong evidence of the differences associated with gender, age, setting, BMI, family history of diabetes, WHO regions, and WB regions in relation to prediabetes. Many other factors are associated with prediabetes in children,^19^, ^22^, ^26^, ^27^ including physical activity, lifestyle, vitamin D, depression, and education level. We could not, however, perform the combined analysis including these factors because of limited data. Despite an extensive search, the selection bias of our study may have led to an overestimation or underestimation of the prevalence of prediabetes at the global level. HbA1c criteria were associated with higher diagnosis rates of prediabetes than fasting plasma glucose and oral glucose tolerance tests in nondiabetic American children and adults^28^, ^29^; however, most of the included studies used only fasting plasma glucose to detect prediabetes, possibly underestimating the prevalence of prediabetes.^30^ Our review combined the prevalence rates of prediabetes for the included studies with different definitions based on the method applied by some meta‐analyses.^31^, ^32^ Subgroup meta‐analysis by diagnosis criteria demonstrates the prevalence difference among diagnostic methods. This may have increased uncertainty intervals because of limited comparability. Sensitivity analysis showed that no single study had a disproportionate influence on the pooled prevalence. Our study established an overall combined estimate of prediabetes prevalence as other studies^31^, ^32^, ^33^ although heterogeneity of included studies is obtrusive. To discover potential sources of heterogeneity, we performed subgroup meta‐analysis in limited groups but found that heterogeneity of the subgroups is also substantial. Because of these limitations, we cannot draw more robust and precise conclusions at this time.

The increase in global prevalence of prediabetes over time is an intermediate stage in the progression of type 2 diabetes mellitus^34^, ^35^ and a major risk factor for cardiovascular disease.^3^, ^36^, ^37^ In a meta‐analysis that included four randomized controlled trials with children, a marked mean reduction in fasting insulin and BMI was observed among participants given metformin (with and without lifestyle intervention) compared to those receiving a placebo after at least 2 months.^38^ A randomized controlled trial has demonstrated that intensive lifestyle modification can improve insulin resistance in obese children with prediabetes.^39^

Intensive lifestyle modification could help to decrease the prevalence and complication of prediabetes. Further longitudinal studies based on large representative samples should identify risk factors and specific biomarkers of prediabetes in childhood so that early targeted intervention strategies can be developed to delay the inexorable increase in prediabetes and related diseases and decrease the cost burden of health care.

## 5 CONCLUSIONS

This study demonstrated that elevated prediabetes prevalence in childhood reaches a considerable level in the world. Intensive lifestyle modification is needed to respond to the prediabetes epidemic globally in the pediatric population.

## AUTHOR CONTRIBUTIONS

Jiang and Hu had full access to all the data in the study and take responsibility for the integrity of the data and the accuracy of the data analysis; Han, Jiang, and Hu conceived and designed the meta‐analysis; Han and Song drafted the manuscript; Han, Song, Ren, Chen, Jiang, and Hu acquired, analyzed, and interpreted the data or revised the manuscript. All authors read and approved of the final manuscript.

## CONFLICT OF INTEREST

No financial conflict or other relationship for each author to be declared.

## Supporting information


**Appendix S1** Supporting Information
