# Supplementary material for: Global prevalence of prediabetes in children and adolescents: A systematic review and meta‐analysis
Source: J Diabetes. 2022 Jul 5;14(7):434–41. doi: 10.1111/1753-0407.13291 (PMC9310043; doi:10.1111/1753-0407.13291)
Supplement: Supplementary file 1 — Appendix S1 Supporting Information [file JDB-14-434-s001.docx]

**Supplementary Online Content**

**eTable 1.** Search Strategy to identify studies reporting the prevalence of prediabetes in children and adolescents

**eTable 2.** The difference between year of publication and year of investigation in the included articles on childhood prediabetes prevalence (n=48)

**eTable 3.** Quality assessment of included studies

**eTable 4.** Characteristics of included studies for prediabetes prevalence in children and adolescents

**eFigure 1.** Pooled prevalence of prediabetes in children and adolescents (n=48)

**eFigure 2.** Leave-1-out sensitivity analysis of the influence of single study on the pooled prevalence of prediabetes in children and adolescents

**This supplementary material has been provided by the authors to give readers additional information about their work.**

**eTable 1. Search Strategy to identify studies reporting the prevalence of prediabetes in children and adolescents**

| Pubmed | |
| --- | --- |
| #1 | "Prediabetic State"[Mesh] OR "Hyperglycemia"[Mesh] OR "Prediabetic State"[Title/Abstract] OR Hyperglycemia[Title/Abstract] OR "impaired fasting glucose"[Title/Abstract] OR "impaired glucose tolerance"[Title/Abstract] OR "pre-diabetes"[Title/Abstract] OR "prediabetes"[Title/Abstract] OR "borderline diabetes"[Title/Abstract] |
| #2 | "prevalence"[Mesh] OR "epidemiology"[Mesh] OR "prevalence"[Title/Abstract] OR "epidemiology"[Title/Abstract] |
| #3 | "adolescent"[Mesh] OR "children"[Title/Abstract] OR "adolescents"[Title/Abstract] |
| #4 | #1 AND #2AND #3 |
| Embase | |
| #1 | 'Hyperglycemia'/exp OR 'Prediabetic State':ab,ti OR Hyperglycemia:ab,ti OR 'impaired fasting glucose':ab,ti OR 'impaired glucose tolerance':ab,ti OR 'pre-diabetes':ab,ti OR prediabetes:ab,ti OR 'borderline diabetes':ab,ti |
| #2 | 'prevalence'/exp OR 'prevalence':ab,ti OR'epidemiology':ab,ti |
| #3 | 'adolescent'/exp OR 'children':ab,ti OR 'adolescents':ab,ti |
| #4 | #1 AND #2AND #3 |
| Web of Science | |
| #1 | TS=('Prediabetic State' OR 'Hyperglycemia' OR 'impaired fasting glucose' OR 'impaired glucose tolerance' OR 'pre-diabetes' OR 'prediabetes' OR 'borderline diabetes') |
| #2 | TS=('prevalence' OR 'epidemiology') |
| #3 | TS= ('children' OR 'adolescents') |
| #4 | #1 AND #2AND #3 |

**eTable 2. The difference between year of publication and year of investigation in the included articles on childhood prediabetes prevalence (n=48)**

| **ID** | **Study** | **Year of publication** | **Year of investigation** | **Time-lag** |
| --- | --- | --- | --- | --- |
| 1 | El-Hazmi,1996 | 1996 | 1993 | 3 |
| 2 | Kim,1999 | 1999 | NA | NA |
| 3 | Fagot-Campagna,2001 | 2001 | 1994 | 7 |
| 4 | Lee,2004 | 2004 | 2000 | 4 |
| 5 | Uçkun-Kitapçi,2004 | 2004 | NA | NA |
| 6 | Whincup,2005 | 2005 | 2000 | 5 |
| 7 | Yamamoto-Kimura,2006 | 2006 | 1998 | 8 |
| 8 | Duncan,2006 | 2006 | 2002 | 4 |
| 9 | Herder,2007 | 2007 | 2005 | 2 |
| 10 | Aradillas-Garcia,2007 | 2007 | NA | NA |
| 11 | Guerrero-Romero,2009 | 2009 | NA | NA |
| 12 | Wahi,2009 | 2009 | NA | NA |
| 13 | Zorzi,2009 | 2009 | NA | NA |
| 14 | Feliciano-Alfonso,2010 | 2010 | 2006 | 4 |
| 15 | Lu,2010 | 2010 | 2006 | 4 |
| 16 | Narayanappa,2011 | 2011 | 2007 | 4 |
| 17 | Aboul Ella,2011 | 2011 | 2008 | 3 |
| 18 | Nsiah-Kumi,2013 | 2013 | 2010 | 3 |
| 19 | Zhang,2013 | 2013 | 2012 | 1 |
| 20 | Sayeed ,2013 | 2013 | NA | NA |
| 21 | Chen,2014 | 2014 | 2011 | 3 |
| 22 | Mamtani,2014 | 2014 | 2012 | 2 |
| 23 | Taranikanti ,2014 | 2014 | NA | NA |
| 24 | Al-Rubeaan,2015 | 2015 | 2009 | 6 |
| 25 | Chahkandi,2015 | 2015 | 2012 | 3 |
| 26 | Jaja,2015 | 2015 | 2013 | 2 |
| 27 | Agbre-Yace,2016 | 2016 | 2013 | 3 |
| 28 | İkinci,2016 | 2016 | NA | NA |
| 29 | Arigbede,2017 | 2017 | 2014 | 3 |
| 30 | Cho,2017 | 2017 | 2014 | 3 |
| 31 | Casapulla,2017 | 2017 | NA | NA |
| 32 | Rodd,2018 | 2018 | 2011 | 7 |
| 33 | Wang,2018 | 2018 | 2014 | 4 |
| 34 | Kloppenborg,2018 | 2018 | NA | NA |
| 35 | Rajani,2018 | 2018 | NA | NA |
| 36 | Yang,2018 | 2018 | NA | NA |
| 37 | Eidkhani,2019 | 2019 | 2005 | 14 |
| 38 | Telo,2019 | 2019 | 2014 | 5 |
| 39 | Arora,2019 | 2019 | 2016 | 3 |
| 40 | Andes,2020 | 2020 | 2016 | 4 |
| 41 | Breyer,2020 | 2020 | 2016 | 4 |
| 42 | Phan,2020 | 2020 | 2018 | 2 |
| 43 | Spurr,2020 | 2020 | NA | NA |
| 44 | Latif, 2020 | 2020 | NA | NA |
| 45 | Akintayo-Usman, 2021 | 2021 | NA | NA |
| 46 | Kim, 2021 | 2021 | 2018 | NA |
| 47 | Shahzad, 2021 | 2021 | 2020 | NA |
| 48 | Mazahery, 2021 | 2021 | 2017 | NA |

Note: NA, not available

**eTable 3. Quality assessment of included studies**

| **ID** | **Study** | **External validity** | | | |  | **Internal validity** | | | | | | **Total** |
| --- | --- | --- | --- | --- | --- | --- | --- | --- | --- | --- | --- | --- | --- |
|  |  | **1^a^** | **2^b^** | **3^c^** | **4^d^** |  | **5^e^** | **6^f^** | **7^g^** | **8^h^** | **9^i^** | **10^j^** |  |
| 1 | El-Hazmi, 1996 | 1 | 1 | 1 | 1 |  | 1 | 1 | 1 | 1 | 0 | 1 | 9 |
| 2 | Kim, 1999 | 0 | 1 | 1 | 0 |  | 1 | 1 | 1 | 1 | 0 | 0 | 6 |
| 3 | Fagot-Campagna, 2001 | 1 | 1 | 1 | 0 |  | 1 | 1 | 1 | 1 | 0 | 1 | 8 |
| 4 | Lee, 2004 | 1 | 1 | 1 | 0 |  | 1 | 1 | 1 | 1 | 0 | 1 | 8 |
| 5 | Uçkun-Kitapçi, 2004 | 0 | 1 | 1 | 1 |  | 1 | 1 | 1 | 1 | 0 | 1 | 8 |
| 6 | Whincup, 2005 | 1 | 1 | 1 | 0 |  | 1 | 1 | 1 | 1 | 0 | 1 | 8 |
| 7 | Yamamoto-Kimura, 2006 | 1 | 1 | 1 | 0 |  | 1 | 1 | 1 | 1 | 0 | 1 | 8 |
| 8 | Duncan, 2006 | 1 | 1 | 1 | 0 |  | 1 | 1 | 1 | 1 | 0 | 0 | 8 |
| 9 | Herder, 2007 | 1 | 1 | 1 | 0 |  | 1 | 1 | 1 | 1 | 1 | 1 | 9 |
| 10 | Aradillas-Garcia, 2007 | 1 | 1 | 1 | 0 |  | 1 | 1 | 1 | 1 | 0 | 1 | 8 |
| 11 | Guerrero-Romero, 2009 | 1 | 1 | 1 | 1 |  | 1 | 1 | 1 | 1 | 0 | 1 | 9 |
| 12 | Wahi, 2009 | 0 | 1 | 1 | 1 |  | 1 | 1 | 1 | 1 | 0 | 1 | 8 |
| 13 | Zorzi, 2009 | 0 | 1 | 1 | 1 |  | 1 | 1 | 1 | 1 | 0 | 1 | 8 |
| 14 | Feliciano-Alfonso, 2010 | 0 | 1 | 1 | 1 |  | 1 | 1 | 1 | 1 | 1 | 1 | 9 |
| 15 | Lu, 2010 | 0 | 1 | 1 | 0 |  | 1 | 1 | 1 | 1 | 1 | 1 | 8 |
| 16 | Narayanappa, 2011 | 0 | 1 | 0 | 0 |  | 1 | 1 | 1 | 1 | 1 | 1 | 7 |
| 17 | Aboul Ella, 2011 | 1 | 1 | 1 | 0 |  | 1 | 1 | 1 | 1 | 0 | 1 | 8 |
| 18 | Nsiah-Kumi, 2013 | 0 | 1 | 1 | 0 |  | 1 | 1 | 1 | 1 | 0 | 1 | 7 |
| 19 | Zhang, 2013 | 0 | 1 | 1 | 0 |  | 1 | 1 | 1 | 1 | 0 | 1 | 7 |
| 20 | Sayeed, 2013 | 1 | 1 | 1 | 0 |  | 1 | 1 | 1 | 1 | 0 | 1 | 8 |
| 21 | Chen, 2014 | 1 | 1 | 1 | 1 |  | 1 | 1 | 1 | 1 | 1 | 1 | 10 |
| 22 | Mamtani, 2014 | 0 | 1 | 1 | 1 |  | 1 | 1 | 1 | 1 | 1 | 1 | 8 |
| 23 | Taranikanti, 2014 | 0 | 1 | 0 | 0 |  | 1 | 1 | 1 | 1 | 0 | 1 | 7 |
| 24 | Al-Rubeaan, 2015 | 1 | 1 | 1 | 1 |  | 1 | 1 | 1 | 1 | 0 | 1 | 9 |
| 25 | Chahkandi, 2015 | 0 | 1 | 1 | 1 |  | 1 | 1 | 1 | 1 | 0 | 1 | 8 |
| 26 | Jaja, 2015 | 0 | 1 | 1 | 1 |  | 1 | 1 | 1 | 1 | 1 | 1 | 9 |
| 27 | Agbre-Yace, 2016 | 0 | 1 | 1 | 1 |  | 1 | 1 | 1 | 1 | 1 | 1 | 9 |
| 28 | İkinci, 2016 | 0 | 1 | 1 | 0 |  | 1 | 1 | 1 | 1 | 0 | 1 | 7 |
| 29 | Arigbede, 2017 | 0 | 1 | 1 | 1 |  | 1 | 1 | 1 | 1 | 1 | 1 | 9 |
| 30 | Cho, 2017 | 1 | 1 | 1 | 1 |  | 1 | 1 | 1 | 1 | 0 | 1 | 9 |
| 31 | Casapulla, 2017 | 0 | 1 | 1 | 0 |  | 1 | 1 | 1 | 1 | 1 | 1 | 8 |
| 32 | Rodd, 2018 | 1 | 1 | 1 | 0 |  | 1 | 1 | 1 | 1 | 0 | 1 | 8 |
| 33 | Wang, 2018 | 1 | 1 | 1 | 0 |  | 1 | 1 | 1 | 1 | 1 | 1 | 9 |
| 34 | Kloppenborg, 2018 | 1 | 1 | 1 | 1 |  | 1 | 1 | 1 | 0 | 0 | 0 | 7 |
| 35 | Rajani, 2018 | 1 | 1 | 1 | 0 |  | 1 | 1 | 1 | 1 | 0 | 1 | 8 |
| 36 | Yang ,2018 | 0 | 1 | 1 | 0 |  | 1 | 1 | 1 | 1 | 0 | 1 | 7 |
| 37 | Eidkhani, 2019 | 0 | 1 | 1 | 1 |  | 1 | 1 | 1 | 1 | 1 | 1 | 9 |
| 38 | Telo, 2019 | 1 | 1 | 1 | 0 |  | 1 | 1 | 1 | 1 | 1 | 1 | 9 |
| 40 | Arora, 2019 | 0 | 1 | 1 | 0 |  | 1 | 1 | 1 | 1 | 0 | 1 | 7 |
| 39 | Andes, 2020 | 1 | 1 | 1 | 1 |  | 1 | 1 | 1 | 1 | 0 | 1 | 9 |
| 41 | Breyer, 2020 | 1 | 1 | 1 | 1 |  | 1 | 1 | 1 | 1 | 0 | 1 | 9 |
| 42 | Phan, 2020 | 1 | 1 | 1 | 0 |  | 1 | 1 | 1 | 1 | 1 | 1 | 9 |
| 43 | Spurr, 2020 | 0 | 1 | 1 | 0 |  | 1 | 1 | 1 | 1 | 0 | 1 | 7 |
| 44 | Latif, 2020 | 0 | 1 | 1 | 1 |  | 1 | 1 | 1 | 1 | 0 | 1 | 8 |
| 45 | Akintayo-Usman, 2021 | 0 | 1 | 1 | 1 |  | 1 | 1 | 1 | 1 | 0 | 1 | 8 |
| 46 | Kim, 2021 | 1 | 1 | 1 | 1 |  | 1 | 1 | 1 | 1 | 0 | 1 | 9 |
| 47 | Shahzad, 2021 | 0 | 1 | 0 | 1 |  | 1 | 1 | 1 | 1 | 1 | 1 | 8 |
| 48 | Mazahery, 2021 | 0 | 1 | 1 | 1 |  | 1 | 1 | 1 | 1 | 1 | 1 | 9 |
| ^a^ Was the study’s target population a close representation of the national population in relation to relevant variables? | | | | | | | | | | | | | |
| ^b^ Was the sampling frame a true or close representation of the target population? | | | | | | | | | | | | | |
| ^c^ Was some form of random selection used to select the sample, OR was a census undertaken? | | | | | | | | | | | | | |
| ^d^ Was the likelihood of nonresponse bias minimal? | | | | | | | | | | | | | |
| ^e^ Were data collected directly from the subjects (as opposed to a proxy)? | | | | | | | | | | | | | |
| ^f^ Was an acceptable case definition used in the study? | | | | | | | | | | | | | |
| ^g^ Was the study instrument that measured the parameter of interest shown to have validity and reliability? | | | | | | | | | | | | | |
| ^h^ Was the same mode of data collection used for all subjects? | | | | | | | | | | | | | |
| ^i^ Was the length of the shortest prevalence period for the parameter of interest appropriate? | | | | | | | | | | | | | |
| ^j^ Were the numerator(s) and denominator(s) for the parameter of interest appropriate? | | | | | | | | | | | | | |

**eTable 4. Characteristics of included studies for prediabetes prevalence in children and adolescents**

| **ID** | **Author** | **Study design** | **Year of Publication** | **Survey time** | **Year** | **Country** | **WHO Region** | **WB Region** | **Data scource** | **Response rate** | **Age range, years** | **Sample size** | **Case** | **Prevalence** | **Definition** | ROB |
| --- | --- | --- | --- | --- | --- | --- | --- | --- | --- | --- | --- | --- | --- | --- | --- | --- |
| 1^1^ | El-Hazmi | Cross-sectional study | 1996 | 1991-1993 | 1993 | Saudi Arabia | EMR | HIC | NA | 95% | 2-14 | 8,762 | 22 | 0.25 | FPG<6.7 mmol/L(IFG)  2h OGTT: 6.7-10.0 mmol/L(IGT) | 9 |
| 2^2^ | Kim | Cross-sectional study | 1999 | NA | NA (1995*) | America | AMR | HIC | NA | 29% | 13-20 | 276 | 8 | 2.90 | FPG: 6.1-6.9mmol/L(IFG)  2h OGTT: 7.8-11.1 mmol/L(IGT) | 6 |
| 3^3^ | Fagot-Campagna | Cross-sectional study | 2001 | 1988–1994 | 1994 | America | AMR | HIC | Third National Health and Nutrition Examination Survey | NA | 12-19 | 2,852 | 22 | 0.77 | Elevated HbA1c>6% | 8 |
| 4^4^ | Lee | Cross-sectional study | 2004 | 1995-2000 | 2000 | America | AMR | HIC | Cherokee Diabetes Study | 52% | 5-19 | 989 | 7 | 0.71 | FPG: 6.1-6.9mmol/L(IFG) | 8 |
| 5^5^ | Uçkun-Kitapçi | Cross-sectional study | 2004 | NA | NA (2000*) | Turkey | EMR | LMIC | The Ankara Adolescent Obesity and Type 2 Diabetes Mellitus Study | 87 | 12-18 | 1,630 | 32 | 1.96 | FPG: 6.1-6.9mmol/L(IFG)，  2h OGTT: 7.8-11.1 mmol/L(IGT) | 8 |
| 6^6^ | Whincup | Cross-sectional study | 2005 | 1998-2000 | 2000 | United Kingdom | EUR | HIC | The Ten Towns Heart Health Study | 62 | 13-16 | 1,338 | 24 | 1.79 | FPG: 6.1-6.9mmol/L(IFG) | 8 |
| 7^7^ | Yamamoto-Kimura | Cross-sectional study | 2006 | 1996-1998 | 1998 | Mexico | AMR | UMIC | NA | 47.3 | 12-16 | 3,121 | 11 | 0.35 | FPG: 6.1-6.9mmol/L(IFG) | 8 |
| 8^8^ | Duncan | Cross-sectional study | 2006 | 1999-2002 | 2002 | America | AMR | HIC | National Health and Nutrition Examination Survey | NA | 12-19 | 1,496 | 178 | 11.90 | FPG: 5.6-6.9mmol/L(IFG) | 8 |
| 9^9^ | Herder | Cross-sectional study | 2007 | 2005 | 2005 | Germany | EUR | HIC | ‘Hauptschulen’ and ‘Sonderschulen’ | NA | 15.5 | 721 | 15 | 2.08 | FPG: 5.6-6.9mmol/L(IFG) | 9 |
| 10^10^ | Aradillas-Garcia | Cross-sectional study | 2007 | NA | NA (2003*) | Mexico | AMR | UMIC | NA | NA | 6-13 | 1,238 | 71 | 5.73 | FPG: 5.6-6.9mmol/L(IFG)  2h OGTT: 7.8-11.1 mmol/L(IGT) |  |
| 11^11^ | Guerrero-Romero | Cross-sectional study | 2009 | NA | NA (2005*) | Mexico | AMR | UMIC | NA | 97 | 6-18 | 1,534 | 360 | 23.47 | FPG: 5.6-6.9mmol/L(IFG)  2h OGTT: 7.8-11.1 mmol/L(IGT) | 8 |
| 12^12^ | Wahi | Cross-sectional study | 2009 | 2006 | 2006 | Canada | AMR | HIC | NA | 100 | 6-18 | 30 | 6 | 20.00 | FPG: 6.1-6.9 mmol/L(IFG)  2h OGTT: 7.8-11.1 mmol/L(IGT) | 9 |
| 13^13^ | Zorzi | Cross-sectional study | 2009 | NA | NA (2005*) | Canada | AMR | HIC | NA | 85 | 6-18 | 192 | 42 | 21.88 | FPG: 5.6-6.9 mmol/L(IFG)  2h OGTT: 7.8-11.1 mmol/L(IGT) | 8 |
| 14^14^ | Feliciano-Alfonso | Cross-sectional study | 2010 | 2005-2006 | 2006 | Colombia | AMR | LMIC | NA | 94 | 15-20 | 249 | 23 | 9.23 | FPG: 5.6-6.9mmol/L(IFG)  2h OGTT: 7.8-11.1 mmol/L(IGT) | 8 |
| 15^15^ | Lu | Cross-sectional study | 2010 | 2006 | 2006 | China | WPR | UMIC | NA | NA | 13-18 | 3,937 | 136 | 3.45 | FPG: 5.6-6.9mmol/L(IFG) | 9 |
| 16^16^ | Narayanappa | Cross-sectional study | 2011 | 2006-2007 | 2007 | India | SEAR | LMIC | NA | NA | 5-10 | 726 | 27 | 3.72 | FPG: 5.6-6.9 mmol/L(IFG) | 8 |
| 17^17^ | Aboul Ella | Cross-sectional study | 2011 | 2000-2008 | 2008 | Egypt | AFR | LMIC | NNI(2000 to 2005) and Egypt Demographic and Health Survey (2008) | NA | 10-18 | 6,018 | 987 | 16.40 | FPG: 5.6-6.9mmol/L(IFG)  2h OGTT: 7.8-11.1 mmol/L(IGT) | 7 |
| 18^18^ | Nsiah-Kumi | Cross-sectional study | 2013 | 2007-2010 | 2010 | America | AMR | HIC | Great Plains tribal diabetes program | NA | 5-18 | 201 | 13 | 6.47 | FPG: 5.6-6.9 mmol/L(IFG)  2h OGTT: 7.8-11.1 mmol/L(IGT) | 7 |
| 19^19^ | Zhang | Cross-sectional study | 2013 | 2010-2012 | 2012 | China | WPR | UMIC | Chun-Miao Project | NA | 0-17 | 3,644 | 24 | 0.66 | FPG: 5.6-6.9 mmol/L(IFG) | 7 |
| 20^20^ | Sayeed | Cross-sectional study | 2013 | NA | NA (2009*) | Bangladesh | EMR | LMIC | NA | NA | 10-18 | 2,152 | 74 | 3.44 | FPG: 6.1-6.9mmol/L(IFG) | 7 |
| 21^21^ | Chen | Cross-sectional study | 2014 | 2010-2011 | 2011 | China | WPR | UMIC | Nutrition and Health Survey in Taiwan | 87% in 2010 and 92% in 2011 | 11-20 | 1,949 | 422 | 21.65 | FPG: 5.6-6.9 mmol/L(IFG) | 8 |
| 22^22^ | Mamtani | Cross-sectional study | 2014 | 2012 | 2012 | Qatar | EMR | HIC | Health assessment program | 99 | 11-18 | 1,694 | 71 | 4.19 | FPG≥5.6 mmol/L  Random PG：≥7.8 mmol/L(IGT) | 7 |
| 23^23^ | Taranikanti | Cross-sectional study | 2014 | NA | NA (2010*) | India | SEAR | LMIC | NA |  | 14-18 | 140 | 10 | 7.14 | FPG:5.6-6.9 mmol/L(IFG) | 8 |
| 24^24^ | Al-Rubeaan | Cross-sectional study | 2015 | 2007-2009 | 2009 | Saudi Arabia | EMR | HIC | SAUDI-DM | 73.48 | 6-18 | 23,523 | 1,053 | 4.48 | FPG: 6.1-6.9 mmol/L(IFG) | 7 |
| 25^25^ | Chahkandi | Cross-sectional study | 2015 | 2012 | 2012 | Iran | EMR | UMIC | NA | 90 | 6-11 | 1,530 | 72 | 4.71 | FPG:5.6-6.9 mmol/L(IFG) | 9 |
| 26^26^ | Jaja | Cross-sectional study | 2015 | 2013 | 2013 | Nigeria | AFR | LMIC | NA | 75 | 10-19 | 880 | 152 | 17.27 | FPG:5.6-6.9 mmol/L(IFG) | 8 |
| 27^27^ | Agbre-Yace | Cross-sectional study | 2016 | 2013 | 2013 | Cote d’Ivoire | AFR | LMIC | NA | NA | 2-19 | 1,572 | 228 | 14.50 | FPG:5.6-6.9 mmol/L(IFG)  2h OGTT:<7.8 mmol/l (IFG) | 9 |
| 28^28^ | İkinci | Cross-sectional study | 2016 | NA | NA (2012*) | Turkey | EMR | LIMC | NA | NA | 9-16 | 805 | 78 | 9.69 | FPG:5.6-6.9 mmol/L(IFG) | 9 |
| 29^29^ | Arigbede | Cross-sectional study | 2017 | 2014 | 2014 | Nigeria | AFR | LMIC | NA | 95.2 | 10-19 | 476 | 19 | 3.99 | FPG:5.6-6.9 mmol/L(IFG) | 7 |
| 30^30^ | Cho | Cross-sectional study | 2017 | 2005-2014 | 2014 | Korea | WPR | HIC | Korea National Health and Nutrition Examination Survey | NA | 10-19 | 6,463,145 | 765,524 | 11.85 | FPG: 5.6–6.9 mmol/L(IFG)  Elevated HbA1c:5.7%–6.5% | 9 |
| 31^31^ | Casapulla | Cross-sectional study | 2017 | 2015 | 2015 | Ecuador | AMR | LMIC | NA | NA | 13-18 | 427 | 53 | 12.41 | Elevated HbA1c:5.7–6.4% | 9 |
| 32^32^ | Rodd | Cross-sectional study | 2018 | 2007-2011 | 2011 | Canada | AMR | HIC | Canadian Health Measures Surveys | NA | 6-19 | 3,449 | 785 | 22.76 | Elevated HbA1c:5.7–6.4% | 8 |
| 33^33^ | Wang | Cross-sectional study | 2018 | 2013-2014 | 2014 | China | WPR | UMIC | NA | NA | 6-17 | 16,434 | 310 | 1.89 | FPG: 5.6–6.9 mmol/L (IFG) | 8 |
| 34^34^ | Kloppenborg | Cross-sectional study | 2018 | 2009-2016 | 2016 | Denmark | EUR | HIC | Danish Childhood Obesity Biobank | 82.3 | 12 | 3,978 | 385 | 9.68 | FPG: 5.6-6.9 mmol/L(IFG) | 9 |
| 35^35^ | Rajani | Cross-sectional study | 2018 | NA | NA (2014*) | India | SEAR | LMIC | NA | NA | 2-15 | 3,864 | 128 | 3.31 | FPG: 5.6–6.9 mmol/L (IFG) | 7 |
| 36^36^ | Yang | Cross-sectional study | 2018 | 2017 | 2017 | China | WPR | UMIC | NA | NA | 6-17 | 7,519 | 737 | 9.80 | FPG: 5.6-6.9 mmol/L(IFG)  Elevated HbA1c: 5.7–6.4% | 8 |
| 37^37^ | Eidkhani | Cohortl study | 2019 | 1999-2005 | 2005 | Iran | EMR | UMIC | NA | 94.9 | 11-19 | 2,998 | 210 | 7.00 | FPG: 5.6-6.9 mmol/L(IFG) | 7 |
| 38^38^ | Telo | Cross-sectional study | 2019 | 2013-2014 | 2014 | Brazil | AMR | UMIC | ERICA | NA | 12-17 | 37,854 | 7646 | 20.20 | FPG: 5.6-6.9 mmol/L(IFG)  Elevated HbA1c: 5.7–6.4% | 9 |
| 39^39^ | Arora | Cross-sectional study | 2019 | 2013-2016 | 2016 | India | SEAR | LMIC | NA | NA | 12-17 | 456 | 23 | 5.09 | FPG: 5.6-6.9 mmol/L(IFG) | 9 |
| 40^40^ | Andes | Cross-sectional study | 2020 | 2005-2016 | 2016 | America | AMR | HIC | National Health and Nutrition Examination Survey | NA | 12-18 | 2,606 | 469 | 18.00 | FPG: 5.6-6.9 mmol/L(IFG)  2h OGTT: 7.8-11.1 mmol/L(IGT)  Elevated HbA1c: 5.7–6.4% | 9 |
| 41^41^ | Breyer | Cohort study | 2020 | 2012-2016 | 2016 | Austria | WPR | HIC | The LEAD study | 96.4 | 6-10 | 393 | 18 | 4.58 | FPG: 5.6-6.9 mmol/L(IFG)  Elevated HbA1c: 5.7–6.4% | 7 |
| 42^42^ | Phan | Cross-sectional study | 2020 | 2018 | 2018 | Vietnam | SEAR | LMIC | NA | NA | 11-14 | 2,880 | 175 | 6.08 | FPG: 5.6-6.9 mmol/L(IFG) | 9 |
| 43^43^ | Spurr | Cross-sectional study | 2020 | 2014-2018 | 2018 | Canada | AMR | HIC | NA | NA | 14-19 | 396 | 10 | 2.53 | Elevated HbA1c: 6.0–6.4% | 9 |
| 44^44^ | Latif | Cross-sectional study | 2020 | NA | NA (2016*) | Saudi Arabia | EMR | HIC | NA | 99 | 18-20 | 297 | 56 | 18.7 | FPG: 5.6-6.9 mmol/L(IFG) | 8 |
| 45^45^ | Akintayo-Usman | Cross-sectional study | 2021 | NA | NA (2017*) | Nigeria | AFR | LMIC | NA | 98.8 | 10-19 | 405 | 38 | 9.4 | FPG: 5.6-6.9 mmol/L(IFG) | 8 |
| 46^46^ | Kim | Cross-sectional study | 2021 | 2007-2018 | 2018 | Korean | WPR | HIC | Korea National Health and Nutrition Examination Survey | 81.2 | 10-18 | 8718 | 690 | 7.9 | FPG: 5.6-6.9 mmol/L(IFG) | 9 |
| 47^47^ | Shahzad | Cross-sectional study | 2021 | 2019-2020 | 2020 | Pakistan | SEAR | LMIC | NA | 93.1 | 16-19 | 351 | 81 | 23.1 | FPG: 5.6-6.9 mmol/L(IFG)  Elevated HbA1c: 5.7–6.4% | 8 |
| 48^48^ | Mazahery | Cross-sectional study | 2021 | 2016-2017 | 2017 | New Zealand | WPR | HIC | Children’s Bone Study | 92 | 8-11 | 451 | 71 | 15.7 | Elevated HbA1c: 5.7–6.4% | 9 |

Note: * Imputed data; NA, not available; WHO, World Health Organization; AFR, African Region; AMR, Region of the Americas; SEAR, South-East Asia Region; EUR, European; Region; EMR, Eastern Mediterranean Region; WPR, Western Pacific Region; WB, World Bank; HIC, high-income countries; UMIC, upper-middle income countries; LMIC, lower-middle income countries.

**
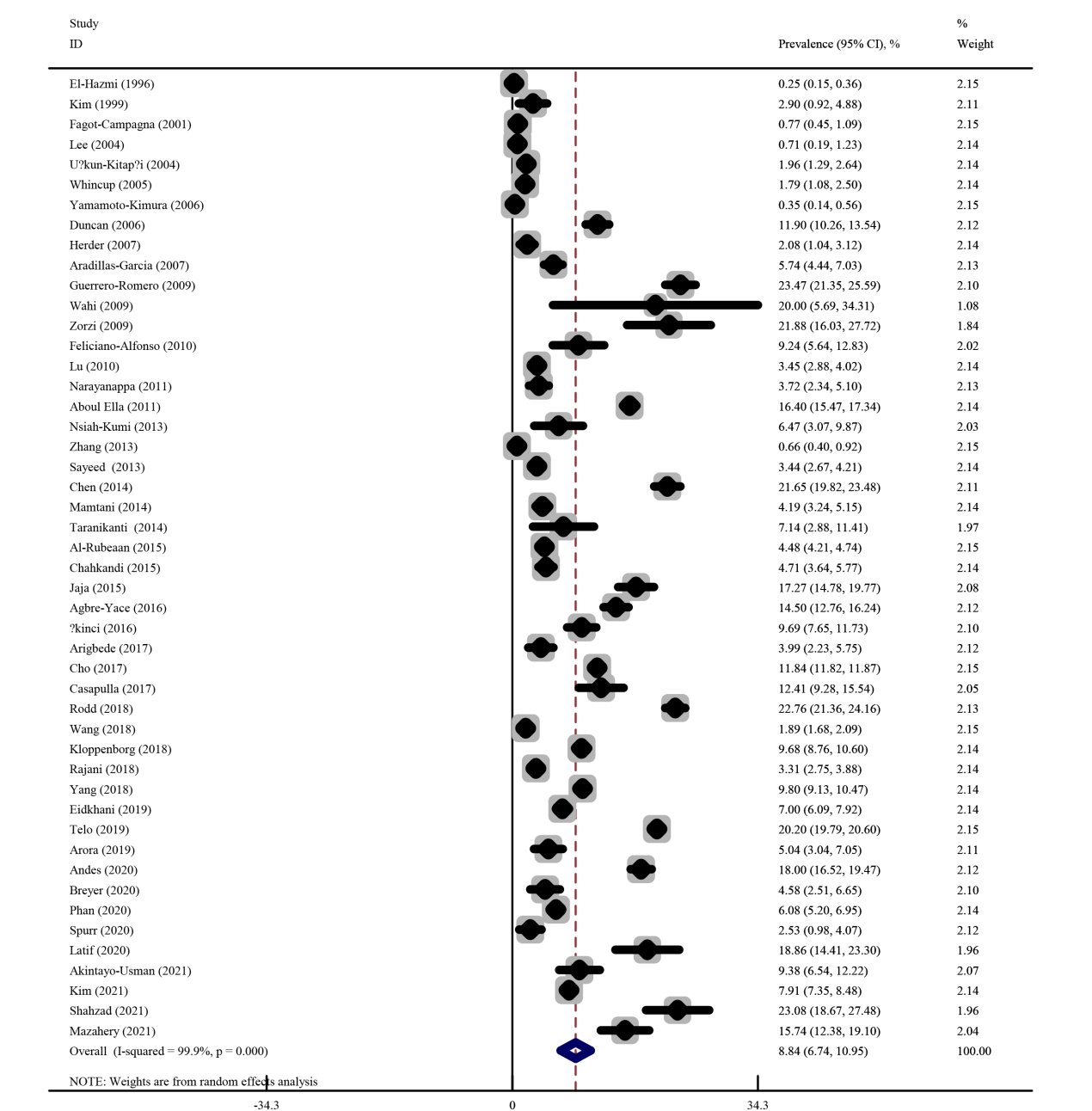
**

**eFigure 1.** Pooled prevalence of prediabetes in children and adolescents (n=48)

**
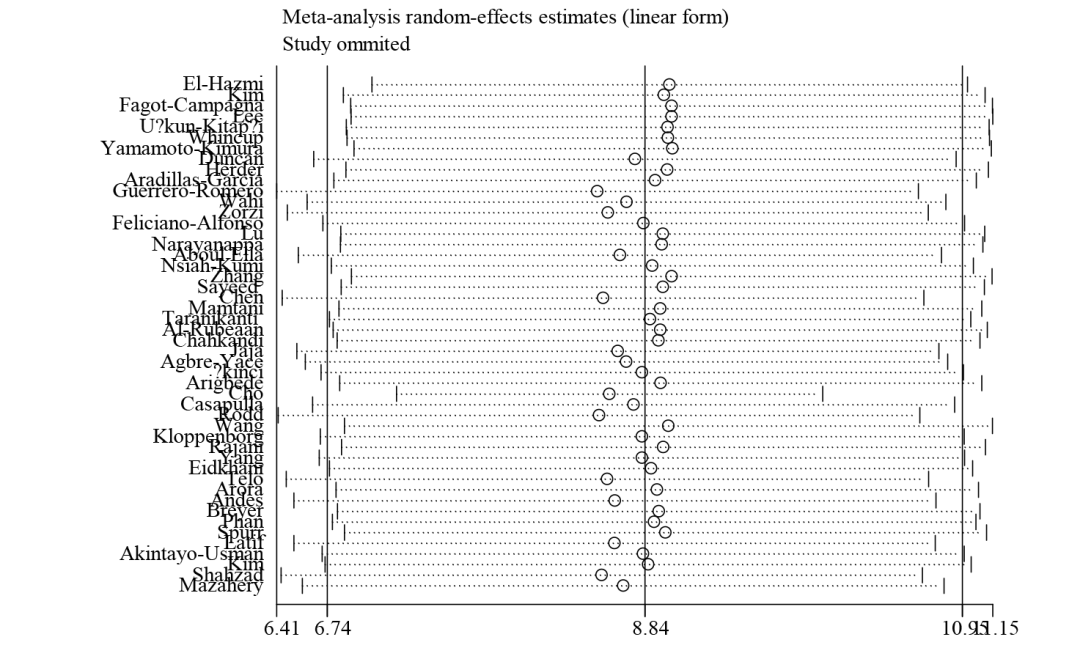
**

**eFigure 2.** Leave-1-out sensitivity analysis of the influence of single study on the pooled prevalence of prediabetes in children and adolescents

**eReferences**

1. El-Hazmi MAF, Warsy AS, Al-Swailem AR, Al-Swailem AM, Sulaimani R, Al-Meshari AA. Diabetes mellitus and impaired glucose tolerance in Saudi Arabia. *Annals of Saudi Medicine.* 1996;16(4):381-385.

2. Kim C, McHugh C, Kwok Y, Smith A. Type 2 diabetes mellitus in Navajo adolescents. *The Western journal of medicine.* 1999;170(4):210-213.

3. Fagot-Campagna A, Saaddine JB, Flegal KM, Beckles GL. Diabetes, impaired fasting glucose, and elevated HbA1c in U.S. adolescents: the Third National Health and Nutrition Examination Survey. *Diabetes care.* 2001;24(5):834-837.

4. Lee ET, Begum M, Wang W, et al. Type 2 diabetes and impaired fasting glucose in American Indians aged 5-40 years: the Cherokee diabetes study. *Annals of epidemiology.* 2004;14(9):696-704.

5. Uçkun-Kitapçi A, Teziç T, Firat S, et al. Obesity and type 2 diabetes mellitus: A population-based study of adolescents. *Journal of Pediatric Endocrinology and Metabolism.* 2004;17(12):1633-1640.

6. Whincup PH, Gilg JA, Owen CG, Odoki K, Alberti KG, Cook DG. British South Asians aged 13-16 years have higher fasting glucose and insulin levels than Europeans. *Diabetic medicine : a journal of the British Diabetic Association.* 2005;22(9):1275-1277.

7. Yamamoto-Kimura L, Posadas-Romero C, Posadas-Sánchez R, Zamora-González J, Cardoso-Saldaña G, Ramírez IM. Prevalence and interrelations of cardiovascular risk factors in urban and rural Mexican adolescents. *Journal of Adolescent Health.* 2006;38(5):591-598.

8. Duncan GE. Prevalence of diabetes and impaired fasting glucose levels among US adolescents: National Health and Nutrition Examination Survey, 1999-2002. *Archives of pediatrics & adolescent medicine.* 2006;160(5):523-528.

9. Herder C, Schmitz-Beuting C, Rathmann W, et al. Prevalence of impaired glucose regulation in German school-leaving students. *International Journal of Obesity.* 2007;31(7):1086-1088.

10. Aradillas-Garcia C, Malacara JM, Garay-Sevilla ME, et al. Prediabetes in rural and urban children in 3 states in Mexico. *Journal of the cardiometabolic syndrome.* 2007;2(1):35-39.

11. Guerrero-Romero F, Violante R, Rodríguez-Morán M. Distribution of fasting plasma glucose and prevalence of impaired fasting glucose, impaired glucose tolerance and type 2 diabetes in the Mexican paediatric population. *Paediatric and perinatal epidemiology.* 2009;23(4):363-369.

12. Wahi G, Zorzi A, Macnab A, Panagiotopoulos C. Prevalence of type 2 diabetes, obesity and the metabolic syndrome among Canadian First Nations children in a remote Pacific coast community. *Paediatrics & child health.* 2009;14(2):79-83.

13. Zorzi A, Wahi G, Macnab AJ, Panagiotopoulos C. Prevalence of impaired glucose tolerance and the components of metabolic syndrome in Canadian Tsimshian Nation youth. *Canadian journal of rural medicine : the official journal of the Society of Rural Physicians of Canada = Journal canadien de la medecine rurale : le journal officiel de la Societe de medecine rurale du Canada.* 2009;14(2):61-67.

14. Feliciano-Alfonso JE, Mendivil CO, Ariza ID, Perez CE. Cardiovascular risk factors and metabolic syndrome in a population of young students from the National University of Colombia. *Revista da Associacao Medica Brasileira (1992).* 2010;56(3):293-298.

15. Lu Q, Yin FZ, Ma CM, et al. Prevalence of impaired fasting glucose and analysis of risk factors in Han adolescents. *J Diabetes Complications.* 2010;24(5):320-324.

16. Narayanappa D, Rajani HS, Mahendrappa KB, Prabhakar AK. Prevalence of prediabetes in school-going children. *Indian pediatrics.* 2011;48(4):295-299.

17. Nebal Abdel Rahman Aboul Ella* DISaMAI. Prevalence of overweight and obesity, and status of chronic non- communicable diseases and some related risk factors among Egyptian adolescents. *Journal of Diabetes and Endocrinology.* 2011;2(4):41-52.

18. Nsiah-Kumi PA, Lasley S, Whiting M, et al. Diabetes, pre-diabetes and insulin resistance screening in Native American children and youth. *International Journal Of Obesity.* 2013;37(4):540-545.

19. Zhang J, Ma YT, Xie X, et al. Prevalence of diabetes and impaired fasting glucose in Uygur children of Xinjiang, China. *Genetics And Molecular Research.* 2013;12(4):5007-5012.

20. M. Abu Sayeed MMR, Nurunnahar Fayzunnessa, Parvin Akter Khanam, Tanjima Begum, Hajera Mahtab, Akhter Banu. Childhood diabetes in a Bangladeshi population. *Journal of Diabetes Mellitus.* 2013;3(1):33-37.

21. Chen CM, Lou MF, Gau BS. Prevalence of impaired fasting glucose and analysis of related factors in Taiwanese adolescents. *Pediatric diabetes.* 2014;15(3):220-228.

22. Mamtani R, Lowenfels AB, Sheikh J, et al. Adolescent prediabetes in a high-risk Middle East country: a cross-sectional study. *JRSM open.* 2014;5(8):2054270414536550-2054270414536550.

23. Taranikanti M, Panda S, Sukanya M, Swamy PN, Khan MS, Tabassum H. Prediabetes in South Indian rural adolescent school students. *Indian journal of physiology and pharmacology.* 2014;58(1):77-80.

24. Al-Rubeaan K. National surveillance for type 1, type 2 diabetes and prediabetes among children and adolescents: a population-based study (SAUDI-DM). *Journal of epidemiology and community health.* 2015;69(11):1045-1051.

25. Chahkandi T, Taheri F, Kazemi T, Bijari B. The Prevalence of Diabetes and Prediabetes Among Elementary School Children in Birjand. *Iranian journal of pediatrics.* 2015;25(1).

26. Jaja T, Oduwole AO, Fetuga B, Abdus-Salam IA. Prevalence of prediabetes in secondary school students in Port Harcourt, Nigeria. *African Journal of Diabetes Medicine.* 2015;23(1):11-14.

27. Agbre-Yace ML, Oyenusi EE, Oduwole AO, Ake MD, Abodo JR. Prevalence of diabetes mellitus among children and adolescents in the district of Abidjan in Cote d'Ivoire: A population-based study. *Journal of diabetes and metabolic disorders.* 2016;15(1).

28. İkinci S, Atak SN, Uysal AR, Köse SK. An estimation of impaired fasting glucose prevalence and related factors: A middle school-based study in children aged 9-16 years. *Nobel Medicus.* 2016;12(2):45-52.

29. Arigbede O, Adeoye I, Jarrett O, Yusuf O. Prediabetes among Nigerian adolescents: A School-based study of the prevalence, risk factors and pattern of fasting blood glucose in Ibadan, Nigeria. *International Journal Of Diabetes In Developing Countries.* 2017;37(4):437-445.

30. Cho EH, Shin D, Cho KH, Hur J. Prevalences and Management of Diabetes and Pre-diabetes among Korean Teenagers and Young Adults: Results from the Korea National Health and Nutrition Examination Survey 2005-2014. *Journal of Korean medical science.* 2017;32(12):1984-1990.

31. Casapulla SL, Howe CA, Mora GR, et al. Cardiometabolic risk factors, metabolic syndrome and pre-diabetes in adolescents in the Sierra region of Ecuador. *Diabetology & metabolic syndrome.* 2017;9.

32. Rodd C, Feely A, Dart A, Sharma A, McGavock J. Biological and socioeconomic determinants of prediabetes in youth: an analysis using 2007 to 2011 Canadian Health Measures Surveys. *Pediatric research.* 2018;84(2):248-253.

33. Wang Z, Zou Z, Wang H, et al. Prevalence and risk factors of impaired fasting glucose and diabetes among Chinese children and adolescents: a national observational study. *British Journal Of Nutrition.* 2018;120(7):813-819.

34. Kloppenborg JT, Fonvig CE, Nielsen TRH, et al. Impaired fasting glucose and the metabolic profile in Danish children and adolescents with normal weight, overweight, or obesity. *Pediatric diabetes.* 2018;19(3):356-365.

35. Rajani HS, Narayanappa D, Jagadish Kumar K, Manjunath VG. Prevalence of prediabetes among traditional population, jenukuruba tribal children in Mysore district. *Indian Journal of Public Health Research and Development.* 2018;9(8):59-62.

36. Yang C, Ding Z, Zhou H, et al. Prevalence of prediabetes by the fasting plasma glucose and HbA1c screening criteria among the children and adolescents of Shenzhen, China. *Journal of diabetes.* 2018.

37. Eidkhani V, Parizadeh D, Hasheminia M, Azizi F, Hadaegh F. Impaired fasting glucose prevalence surge among Iranian adolescents in a decade: The Tehran Lipid and Glucose Study. *Pediatric diabetes.* 2019;20(8):1064-1071.

38. Telo GH, Cureau FV, Szklo M, Bloch KV, Schaan BD. Prevalence of type 2 diabetes among adolescents in Brazil: Findings from Study of Cardiovascular Risk in Adolescents (ERICA). *Pediatric diabetes.* 2019;20(4):389-396.

39. Arora B, Patel SS, Saboo BD. The prevalence of prediabetes and associated conditions in Ahmedabad population. *International Journal of Diabetes in Developing Countries.* 2019.

40. Andes LJ, Cheng YJ, Rolka DB, Gregg EW, Imperatore G. Prevalence of Prediabetes Among Adolescents and Young Adults in the United States, 2005-2016. *JAMA pediatrics.* 2020; 174(2):e194498.

41. Breyer MK, Ofenheimer A, Altziebler J, et al. Marked differences in prediabetes- and diabetes-associated comorbidities between men and women-Epidemiological results from a general population-based cohort aged 6-80 years-The LEAD (Lung, hEart, sociAl, boDy) study. *European journal of clinical investigation.* 2020;50(3):e13207.

42. Phan DH, Do VV, Khuong LQ, Nguyen HT, Minh HV. Prevalence of Diabetes and Prediabetes among Children Aged 11-14 Years Old in Vietnam. *Journal of diabetes research.* 2020;2020:7573491.

43. Spurr S, Bally J, Bullin C, Allan D, McNair E. The prevalence of undiagnosed Prediabetes/type 2 diabetes, prehypertension/hypertension and obesity among ethnic groups of adolescents in Western Canada. *BMC pediatrics.* 2020;20(1):31.

44. Latif R, Rafique N. Prevalence and Risk Factors of Prediabetes in Young Saudi Females in a University Setting. *Ethiop J Health Sci*. 2020;30(6):929-940.

45. Akintayo-Usman NO, Okanlawon FA, Usman SO. Prevalence of pre-diabetes and risk factors among secondary school adolescents in Osogbo Local Government Area, Osun State, Nigeria. *Afr Health Sci*. 2021;21(3):1301-1309.

46. Kim JH, Lim JS. Trends of Diabetes and Prediabetes Prevalence among Korean Adolescents From 2007 to 2018. *Journal of Korean Medical Science*. 2021;36(17).

47. Shahzad F, Ishaque A, Saleem F. Prediabetes in adolescents — An emerging epidemic — A cross-sectional survey of medical students at a public university, Quetta, Pakistan. *Journal of the Pakistan Medical Association*. 2021;71(5):1438-1441.

48. Mazahery H, Gammon CS, Lawgun D, Conlon CA, Beck KL, von Hurst PR. Pre-diabetes prevalence and associated factors in New Zealand school children: a cross-sectional study. *N Z Med J*. 2021;134(1531):76-90.
